# Peer Review for “Identification of COVID-19–Associated Hepatitis in Children as an Emerging Complication in the Wake of SARS-CoV-2 Infections: Ambispective Observational Study”

**DOI:** 10.2196/59596

**Published:** 2024-10-11

**Authors:** Mario Coccia

**Affiliations:** 1 National Research Council of Italy, Rome, Italy

**Keywords:** COVID-19, coronavirus, SARS-CoV-2, liver, hepatic, hepatitis, child, children, pediatric, pediatrics, retrospective, observational, jaundice, youth, inflammatory, inflammation

*This is the peer-review report for “Identification of COVID-19–Associated Hepatitis in Children as an Emerging Complication in the Wake of SARS-CoV-2 Infections: Ambispective Observational Study.”*

## Round 1 Review

COVID-19–associated hepatitis in children (CAH-C) during the second wave of SARS-CoV-2 infections in Central India: is it a complication or transient phenomenon?

The topics of this paper [1] are interesting but the results are not clear and robust. The structure and content must be revised, and the results have to be better explained by the authors before being reconsidered for publication.

### Title and Abstract

The title has to be shorter.

The abstract has to clarify the goal, empirical results, and health and social policy for children to deal with this disease.

### Introduction

The introduction has to better clarify the research questions of this study and provide more theoretical background. The authors have to better describe the different sources of transmission dynamics of COVID-19 (eg, climate, air pollution, etc) and risk factors in society, which can accelerate the diffusion of this novel coronavirus in the environment and the emergence of this hepatic disease in children. After that, they can focus on the topics of this study to provide a correct analysis for fruitful discussion (see suggested readings that must be all read and used in the text).

### Methods

The methods of this study are not clear. The *Materials and Methods* section must be restructured with following 3 sections only and in the same order:

- Sample and data
- Measures of variables
- Data analysis procedure

Inclusion and exclusion criteria can be better clarified for readers in a table.

The authors must avoid a lot of subheadings that create fragmentation and confusion. If necessary, they can use bullet points (same comment for the *Results* section and all sections).

### Results

Titles of tables must always indicate the period under study to be clear.

For Table 1, insert a note to clarify the acronyms. The results are not clear, because they can be stronger with a comparative analysis.

…

The emergence of hepatitis in children can be investigated while considering the interaction of COVID-19 with air pollution in the environment and other factors. These aspects have to be discussed, analyzing hepatitis in a spatial dimension and considering pollution.

### Discussion

First, the authors have to synthesize the main results in a simple table to ensure clarity for readers and then show what this study adds compared to other studies.

The conclusion has to be an autonomous section. The conclusion does not have to be a summary, but the authors have to focus on manifold limitations of this study and provide suggestions of health, crisis management, and social policy to cope with COVID-19 and hepatitis in children.

Overall, then, the paper is interesting, but the structure is confusing, and the results are not strong without a comparative analysis. The theoretical framework is weak, and some results create confusion…the structure of the paper has to be improved. The study design, discussion, and presentation of results have to be clarified using the suggested comments.

I strongly suggest improving the paper by using all the comments (suggested papers have been included for the authors to read, and all should be used), which I will verify in depth, and then maybe it can be considered. If the paper is not improved as suggested, it will be dismissed.

Suggested readings of relevant papers that have to be read and all inserted in the text and references to improve, extend, and enrich the theory and discussion of results for implications of pandemic and postpandemic crisis management:

- Peters AL, Kim S, Mourya R, et al. Recent increase in incidence of severe acute hepatitis of unknown etiology in children is associated with infection with adenovirus and other nonhepatotropic viruses. J Pediatr. Aug 2023;259:113439. [doi: 10.1016/j.jpeds.2023.113439] [Medline: 37088181]
- Zeng G, Huang J. The recent outbreak of acute severe hepatitis in children of unknown origin. J Hepatol. Oct 2022;77(4):1213-121. [doi: 10.1016/j.jhep.2022.05.017] [Medline: 35644435]
- Coccia M. Sources, diffusion and prediction in COVID-19 pandemic: lessons learned to face next health emergency. AIMS Public Health. Mar 2, 2023;10(1):145-168. [doi: 10.3934/publichealth.2023012] [Medline: 37063362]
- Brüssow H. Non-A to E hepatitis in children: detecting a novel viral epidemic during the COVID-19 pandemic. Microb Biotechnol. Oct 2023;16(10):1879-1887. [doi: 10.1111/1751-7915.14329] [Medline: 37602673]
- Núñez-Delgado A, Bontempi E, Coccia M, Kumar M, Farkas K, Domingo JL. SARS-CoV-2 and other pathogenic microorganisms in the environment. Environ Res. Oct 2021;201:111606. [doi: 10.1016/j.envres.2021.111606] [Medline: 34181924]
- Gates S, Andreani J, Dewar R, et al. Postpandemic rebound of adeno-associated virus type 2 (AAV2) infections temporally associated with an outbreak of unexplained severe acute hepatitis in children in the United Kingdom. J Med Virol. Jul 2023;95(7):e28921. [doi: 10.1002/jmv.28921] [Medline: 37403889]
- Coccia M. Factors determining the diffusion of COVID-19 and suggested strategy to prevent future accelerated viral infectivity similar to COVID. Sci Total Environ. Aug 10, 2020;729:138474. [doi: 10.1016/j.scitotenv.2020.138474] [Medline: 32498152]
- Leiskau C, Tsaka S, Meyer-Ruhnke L, et al. Acute severe non-A-E-hepatitis of unknown origin in children – a 30-year retrospective observational study from north-west Germany. J Hepatol. May 2023;78(5):971-978. [doi: 10.1016/j.jhep.2022.12.012] [Medline: 36572350]
- Coccia M. How do low wind speeds and high levels of air pollution support the spread of COVID-19? Atmos Pollut Res. Jan 2021;12(1):437-445. [doi: 10.1016/j.apr.2020.10.002] [Medline: 33046960]
- Cooper S, Waisbourd-Zinman O. Reply to: severe hepatitis in children likely caused by HAdV-41 following SARS-CoV-2. J Pediatr Gastroenterol Nutr. Mar 1, 2023;76(3):e70. [doi: 10.1097/MPG.0000000000003689] [Medline: 36574220]
- Coccia M. Pandemic prevention: lessons from COVID-19. Encyclopedia. May 31, 2021;1(2):433-444. [doi: 10.3390/encyclopedia1020036]
- Mohammed FS, Karnsakul W, Mohammed S, Russo MW. Severe hepatitis in children likely caused by HAdV-41 following SARS-CoV-2 induced mitochondrial permeability transition. J Pediatr Gastroenterol Nutr. Mar 1, 2024;76(3):e69. [doi: 10.1097/MPG.0000000000003688] [Medline: 36574266]

## Abbreviations

CAH-C: COVID-19–associated hepatitis in children
